# A Polish Study on the Influence of Food Neophobia in Children (10–12 Years Old) on the Intake of Vegetables and Fruits

**DOI:** 10.3390/nu9060563

**Published:** 2017-06-02

**Authors:** Dominika Guzek, Dominika Głąbska, Ewa Lange, Marzena Jezewska-Zychowicz

**Affiliations:** 1 Department of Organization and Consumption Economics, Faculty of Human Nutrition and Consumer Sciences, Warsaw University of Life Sciences (SGGW-WULS), 159C Nowoursynowska Street, 02-787 Warsaw, Poland; marzena_jezewska_zychowicz@sggw.pl; 2 Department of Dietetics, Faculty of Human Nutrition and Consumer Sciences, Warsaw University of Life Sciences (SGGW-WULS), 159C Nowoursynowska Street, 02-787 Warsaw, Poland; dominika_glabska@sggw.pl (D.G.); ewa_lange@sggw.pl (E.L.)

**Keywords:** food neophobia, Food Neophobia Scale (FNS), children, fruits, vegetables, juices

## Abstract

Adhering to the recommended intake of fruits and vegetables is an important habit that should be inculcated in children, whereas food neophobia is indicated as one of the most important factors creating food preferences that may interfere. The aim of the presented study was to analyze the association between the food neophobia level and the intake of fruits and vegetables in children aged 10–12 years. The study was conducted among a group of 163 children (78 girls and 85 boys). The assessment of the food neophobia level was based on the Food Neophobia Scale (FNS) questionnaire and the assessment of the fruit and vegetable intake was based on the food frequency questionnaire. A negative correlation between the food neophobia level and the vegetable intake was observed both for girls (*p* = 0.032; R = −0.2432) and for boys (*p* = 0.004; R = −0.3071), whereas for girls differences in vegetable intake were observed also between various food neophobia categories (*p* = 0.0144). It may be concluded that children with higher food neophobia level are characterized by lower vegetable intake than children with lower food neophobia level. For fruits and juices of fruits and vegetables, associations with food neophobia level were not observed.

## 1. Introduction

Inadequate intake of fruits and vegetables is indicated as a reason for 6.7 million deaths worldwide each year, which was estimated for the year 2010 [1]. The World Health Organization indicated in a report [2] that, if consumed in the recommended amounts, fruits and vegetables reduce the risk of noncommunicable diseases, including coronary heart disease, stroke, and some types of cancers. 

Especially in the case of children, vegetable intake is perceived as beneficial, and consuming them in every meal is a positive behavior that may contribute to healthier dietary patterns [3]. Moreover, it was confirmed in a Polish study on a group of girls that the pattern associated with high fruit and vegetable intake was connected with greater restrictions in the intake of products high in sugar, fat, and starch [4].

In spite of the beneficial effects of fruits and vegetables, in a study of 52 countries taking part in the World Health Survey (2002–2003), the intake was stated to be lower than the lowest recommended number of five servings per day (80 g of fruits/vegetables per serving) for 77.6% of men and 78.4% of women from all the countries [5]. Similarly, in the Child and Adolescent Health Surveys (KiGGS wave 1), it was found that in the age group of 3–17 years, girls consumed 2.7 and boys consumed 2.4 servings of fruits and vegetables per day, whereas only 12.2% of girls and 9.4% of boys consumed five servings per day [6]. 

It is emphasized that even higher fruit and vegetable intake must be considered, as the World Health Organization recommends 600 g (7.5 servings) per day in adults and 480 g (6 servings) per day in children aged 5–14 years [7]. Achieving the recommended intake of fruits and vegetables in children is a challenge. On the one hand, established children’s dietary patterns predict adulthood dietary patterns, but on the other, compared with adults, there are additional factors influencing children’s fruit and vegetable intake [2]. Considering both factors, parental role modeling is important, as it is a significant predictor of children’s dietary patterns [8]. In general, it may be indicated that the two determining factors, not observed in the case of adults, are parental intake and home accessibility, while the other most prominent determinants indicated for children are gender, age, socioeconomic position of the family, and preferences [9]. 

Among all the determinants of food choices indicated for children, only their preferences are directly dependent on them. However, children typically prefer familiar, bland, and sweet products, whereas in the case of unknown food products, aversion may occur [10]. Food neophobia is indicated as the most important factor creating food preferences in the case of younger children [11], but also, in the case of children aged 10–11 years, it is emphasized that they should be introduced to unfamiliar fruits and vegetables in order to increase their taste preferences [12].

The aim of the presented study was to analyze the association between the food neophobia level and the intake of fruits and vegetables in children aged 10–12 years.

## 2. Materials and Methods

### 2.1. Ethics Approval 

The study was conducted according to the guidelines laid down in the Declaration of Helsinki, and all the procedures involving human subjects were approved by the Ethics Committee of the Faculty of Human Nutrition and Consumer Sciences of the Warsaw University of Life Sciences (SGGW-WULS) in Warsaw, Poland (No. 10/2016; 12.12.2016).

### 2.2. Study Participants

The study group was recruited in the group of children aged 10–12 years from Warsaw, who participated in the scientific nutrition workshops for children conducted in the Dietary Outpatient Clinic of the Faculty of Human Nutrition and Consumer Sciences of the SGGW-WULS. Inclusion criteria were children aged 10–12 years, not suffering from any developmental disorder affecting intellectual abilities, including various intellectual or cognitive deficits. The information about nutritional workshops for children aged 10–12 years was placed on the web page, and 258 children were signed up for the workshops by their parents. The workshops’ participants were proposed to take part in the study for analyzing the association between food neophobia level and fruit and vegetable intake. One hundred and seventy-five children agreed to participate, and their parents or legal guardians also provided written consent to participate. The exclusion criterion was nonprovision of written consent to participate.

**Figure 1:**
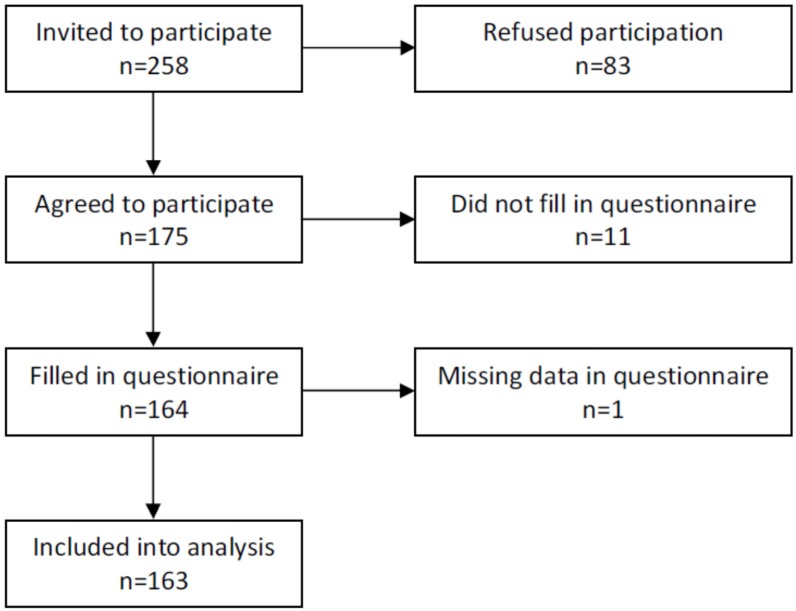
Study design and number of participants.

The participants were asked to fill in two questionnaires: the Food Neophobia Scale (FNS) questionnaire by Pliner and Hobden [13] and the food frequency questionnaire consisting of 31 questions about various groups of products. Because not all participants filled in both questionnaires and due to some missing data, 163 participants (78 girls and 85 boys) were included in the final analysis. The study design and number of participants are presented in Figure 1.

### 2.3. Assessment of the Food Neophobia Level

The FNS questionnaire was applied in order to assess the food neophobia level in the analyzed individuals. Each individual received the list of sentences and was asked to rate his or her level of agreement with each sentence, using one of the seven categories of answer (scale from strongly disagree to strongly agree). Because the 10-item FNS questionnaire of Pliner and Hobden [13] includes five positive items (indicating neophilic individuals) and five negative items (indicating neophobic individuals), negative responses were reversed during the analysis of data [14]. In the present group, Cronbach’s alpha was at a respectable level (0.78; *n* = 163), and the same respectable level was observed for the group of boys (0.78; *n* = 78), whereas for the group of girls, it was stated to be very good (0.85; *n* = 85), indicating good internal consistency [15].

The calculated food neophobia level ranged from 10 to 70 and, on the basis of the level, the participants were divided into three tertiles characterized by various food neophobia levels: low values (first tertile) are attributed to a low neophobia level (neophilic), medium values (second tertile) are attributed to a medium neophobia level, and high values (third tertile) are attributed to a high neophobia level (neophobic) [16].

### 2.4. Assessment of the Fruit and Vegetable Intake

The food frequency questionnaire was applied in order to assess the typical intake of fruits and vegetables as well as fruit and vegetable juices among the analyzed individuals. In order to reduce overestimation due to high self-appraisal, the respondents were asked not only about fruits and vegetables, and fruit and vegetable juices, but also about all the most important food products groups, without giving any information as to which items would be assessed. Potatoes were also categorized as a separate group to avoid misinterpreting them as vegetables. The applied questionnaire was previously positively validated on the basis of a Bland-Altman plot in a group of 172 children.

Each individual received the list of food products groups with specified serving size and was asked about the exact number of servings (calculated not only in integers, but also decimal parts) of the products specified in the questionnaire consumed per day/week/month (depending on the product), according the methodology described previously [17]. In the case of fruits and vegetables, participants were asked to specify the number of servings consumed per day, whereas in the case of fruit and vegetable juices, they were asked to specify the number of servings consumed per week. In the case of fruits and vegetables, the described serving size was 100 g, so the numbers of servings were multiplied by 100 g to obtain the typical intake value per day. In the case of fruit and vegetable juices, the described serving size was 250 g, so the number of servings was multiplied by 250 g and divided by seven (days) to obtain a typical intake value per day. The serving sizes were not only expressed in terms of grams, but also described using typical household measures.

### 2.5. Statistical Analysis

The obtained data are presented as means ± standard deviation (SD) with minimum, maximum, and median values. The distributions of the analyzed factors were verified by using the Shapiro-Wilk test. Internal reliability of the FNS for the group was tested using the Cronbach’s alpha coefficient. Differences between groups were identified by using the U Mann-Whitney test and Kruskal-Wallis ANOVA test (applied for nonparametric distribution). Analyses of the correlations were verified by using Spearman’s rank correlation coefficient (applied for nonparametric distribution). 

The accepted level of significance was set at *p* ≤ 0.05. Statistical analysis was conducted using Statistica software version 8.0 (StatSoft Inc., Tulsa, OK, USA).

## 3. Results

The assessment of fruit intake observed for the analyzed groups of girls and boys in various food neophobia categories is presented in Table 1. No differences in fruit intake in the groups of children characterized by various neophobia levels were stated for both girls and boys. Also, no differences in fruit intake between girls and boys from the same neophobia level were observed. 

The assessment of vegetable intake observed for the analyzed groups of girls and boys in various food neophobia categories is presented in Table 2. No differences in vegetable intake in the group of boys characterized by various neophobia levels were found. In the group of girls, it was found that the differences in vegetable intake observed between various food neophobia categories were statistically significant (*p* = 0.0144). At the same time, no differences in vegetable intake between girls and boys from the same neophobia level were observed. 

The assessment of fruit and vegetable juice intake observed for the analyzed groups of girls and boys in various food neophobia categories is presented in Table 3. No differences in fruit and vegetable juice intake in the groups of children characterized by various neophobia levels were stated for both girls and boys. Also, no differences in fruit and vegetable juice intake between girls and boys from the same neophobia level were observed. 

To verify the differences in fruit and vegetable intake observed between girls of various food neophobia categories, analysis of correlation between the food neophobia level and the intake of fruits, vegetables, and fruit and vegetable juices was conducted (Table 4). It was confirmed that neither for fruits nor for fruit and vegetable juices, the association between food neophobia level and intake exists, for both girls and boys. Simultaneously, the previously indicated association for vegetables was proven. It was stated that the negative correlation between the food neophobia level and vegetable intake exists in both girls (*p* = 0.032; R = −0.2432) and boys (*p* = 0.004; R = −0.3071); thus, it may be indicated that children with a higher neophobia level are characterized by lower vegetable intake than children with a lower neophobia level.

## 4. Discussion

Considering the low fruit and vegetable intake in children, indicating the factors that may influence it as well as suggesting the possible ways to overcome the observed trend may be crucial to improve the nutritional value of diet by increasing its variety [18]. Food neophobia (defined as reluctance or avoidance of unknown food products) and pickiness (defined as consuming an inadequate variety of food products, due to the rejection of substantial number of them) are indicated as the most important factors that may cause low vegetable intake [19]. Pickiness is not always reasoned by food neophobia [20] but, in the case of food neophobia, pickiness of unknown food products is common [21]. 

Food neophobia in children may be associated not only with pickiness, but also with unwillingness to even try unfamiliar food products, which might result in following an improperly balanced diet [18]. Because of food neophobia in adolescence, neophobic behaviors may be transferred to adulthood [22], as it was indicated that such behaviors often remain stable from the age of 13 years to adulthood [23]. At the same time, the age of 9 years is indicated as critical because before that age, the development of food behaviors takes place [24]. Taking this into account, the analysis of food neophobia, its determinants, and consequences among the group of children aged 10–12 years is of a great value, as at such an age there is still a possibility of creating preferences and to trying to reduce the level of neophobic behaviors. 

It needs to be emphasized that, in spite of the fact that in preschool children (early childhood) parents can influence dietary patterns [25], in the late childhood, parental influence is reduced in comparison with early childhood, which is associated inter alia with not participating in family dinners [26]. However, the influence of peers is also an important factor, which was proven in the case of vegetable choices, as it was observed that eating vegetables with peers was associated not only with choosing nonpreferred ones, while peers did so, but also with changing preferences [27].

Fruit and vegetable choices in general are associated with children preferences and accessibility [14], which are related to the factors associated not only with the family but also with the country. In the Polish population, it is indicated that national traditions and customs may influence vegetable intake, as they may, for example, cause higher cabbage intake, which is associated with a number of traditional recipes of cabbage dishes that are commonly consumed [28]. However, among the most preferred and most frequently consumed vegetables in Poland in the group of school children are carrots, cucumbers, radishes, and tomatoes; among the most preferred fruits are strawberries, tangerines, oranges, and blueberries, whereas among the most frequently consumed fruits are apples, tangerines, bananas, and oranges [29]. At the same time, in a group of Spanish children and young people, among the most preferred fruits and vegetables are similar products such as apples, bananas, carrots, tomatoes, and lettuce [30].

Vegetable intake is important in the context of food neophobia, as it concerns mainly fruits and vegetables [31]. Moreover, it is indicated that in general, for children, vegetables are characterized by a lower acceptance level than fruits [32], and is even observed to be the lowest among the acceptance levels for all the food product groups [33]. This may partly explain the results of the present study, and the fact that vegetable intake was more prone than fruit intake to be reduced in the case of children characterized by a high food neophobia level. This may result from the fact that vegetables are not as sweet as fruits, and sometimes even have a bitter taste [34], and thus may be subjected to natural rejection evolved as an adaptive safeguard reaction to the potential toxicity of food products [35]. However, in the case of the analyzed group, who have had more experiences with a variety of food products compared to younger children, factors other than naturally evolved reactions must be rather taken into account.

Among the factors influencing vegetable intake, food neophobia and pickiness have been indicated as the important ones. In the study of Galloway et al. [36], it was indicated that girls characterized by high food neophobia and pickiness had higher vegetable intake than those characterized by low food neophobia and pickiness. However, in the mentioned study, in a comparison between the groups of girls with high food neophobia accompanied by low pickiness and those with low food neophobia accompanied by high pickiness, no differences between groups were indicated [36]. In the case of the present study, a similar association was observed; however, new conclusions may be formulated. It may be supposed that food neophobia not only influences vegetable intake when combined with pickiness but, in the population of Polish children, may also be the strong independent factor that may influence it alone. However, to draw more general conclusions, further studies should be conducted also in countries other than Poland. 

Another new insight into the area of food neophobia is associated with the fact that, in the study of Galloway et al. [36], only a group of girls was analyzed. It is well known that among girls and boys, food preferences may differ, wherein girls are characterized by a higher general preference of fruits and vegetables compared to boys [33]. Moreover, it is indicated that not only boys are characterized by a higher food neophobia level than girls, but also adult males than females [37]. However, concerning food neophobia, in the majority of studies on the association between food neophobia level and intake of fruits and vegetables in children, the authors analyze data for the combined groups of boys and girls [38,39,40,41,42], whereas only a few studies present data for boys and girls separately or for only one gender [33,36,43].

Although the attitude toward food products may differ between boys and girls, analyzing them as one combined group may result in changing observed associations. For example, a study by Falciglia et al. [18] reported that no relationship between food neophobia level and vegetable intake was observed, however, this finding may have resulted from analyzing boys and girls together. 

At the same time, in the study of Tsuji et al. [43], a high food neophobia level in Japanese boys was associated with low vegetable intake, which was not observed in the group of girls. On the one hand, it is in agreement with the results of Galloway et al. [36], as in the mentioned study not a high food neophobia level alone, but only that combined with a high level of pickiness was associated with low vegetable intake in girls. However, on the other hand, it should be mentioned that vegetable intake in the Japanese diet is different from that in the Western diet, while, for example, a higher level of soya intake must be considered [43].

The results of the conducted study indicated that both girls and boys, aged 10–12 years, with a higher food neophobia level may be characterized by lower vegetable intake than children with a lower food neophobia level. It must also be emphasized that, in the case of girls, the association was stronger than that found in boys, as was observed in both the analysis of correlations and comparison between the groups of various food neophobia levels. 

Such an observation may be useful for public health purposes. In order to obtain the recommended vegetable intake in children, the reduction of the food neophobia levels may be essential. It may be achieved by taste education and food exposures, which may lead to creating new dietary habits [44,45]. However, it is also observed that implementing taste education and food exposures has produced contradictory results; for vegetables, either a successful education [27] or lack of success was observed [45] in various studies. Taking this into account, not only the association between food neophobia level and vegetable intake must be assessed, but also the most efficient ways of education must be analyzed in order to achieve the reduction of food neophobia levels.

In spite of the promising results of this study, some limitations exist. On the one hand, the general heterogeneity of the analyzed group is positive, as it represents a group representative of the Polish population of children aged 10–12 years more realistically than a homogeneous group. However, on the other hand, it may be argued that in a heterogeneous group, the observed associations may be influenced by the existing variations between individuals. Moreover, in spite of the fact that the food frequency questionnaire is one the most commonly applied methods in dietary research, this tool is also limited by specific error associated with fact that it is a retrospective method, based on the memory of respondents [46]. However, the applied food frequency questionnaire was a previously validated and comprehensive questionnaire, in which respondents were asked not only about fruit and vegetable intake, but also about all food products groups, without specifying which items would be assessed. Applying a self-administrated questionnaire is also a well-known method to reduce a social desirability bias, and is thus considered better than other methods of questionnaire administration. Such an approach may reduce the effect of desirable bias, but it is still not completely eliminated [47]. Taking into account the aim of this study, it may be indicated that the systematic errors resulting from the applied method did not influence the association between the food neophobia level and the observed intake of fruits and vegetables in children aged 10–12 years. 

Simultaneously, it must be emphasized that the varying results of the studies conducted worldwide may result from differences in the applied methodology, as well as from cultural differences between children from analyzed countries. The previously indicated differences of the most commonly chosen fruits and vegetables between Asian and European countries, as well as the indicated similarities of the most commonly chosen fruits and vegetables between European countries, are confirmed by the broad data from international comparisons. Taking into account the data from 2013, it must be indicated that fruit intake in Poland was similar to those in countries in the geographical proximity, such as Czech Republic, Slovakia, Hungary, Latvia, Ukraine, Republic of Moldavia, Romania, and the Russian Federation [48], and vegetable intake in Poland was also similar to those in countries such as Hungary, Lithuania, Slovenia, Serbia, Croatia, Republic of Moldavia, as well as Germany and Austria [48]. Given the abovementioned data, it must be emphasized that the obtained results could be useful to compare with results from other countries characterized by similar intake. Still, further studies are also recommended in order to confirm these observations in other European or non-European countries.

## 5. Conclusions

1. Children, aged 10–12 years, with a higher food neophobia level may be characterized by lower vegetable intake than children with a lower neophobia level.
2. The association between food neophobia level and vegetable intake in the case of girls aged 10–12 years seems to be stronger than that in the case of boys.
3. In the case of children aged 10–12 years, in order to increase the vegetable intake, education must be conducted to achieve a reduction in the food neophobia level. However, further studies are also needed.

## Figures and Tables

**Table 1:** Fruit intake (g/day) for boys and girls in Food Neophobia Scale categories—mean ± SD, as well as median, minimum, and maximum values are presented, and compared between genders and between food neophobia categories.

Food Neophobia Category (Tertile of Food Neophobia Scale)	Girls; *n* = 78		Boys; *n* = 85		*p*-Value
	Mean ± SD	Median (Minimum–Maximum)	Mean ± SD	Median (Minimum–Maximum)	
Low (first)	198.1 ± 122.0	200.0 * (0.0–500.0)	202.1 ± 156.2	200.0 * (10.0–600.0)	0.7077
Medium (second)	180.8 ± 99.1	175.0 * (50.0–400.0)	187.9 ± 108.3	200.0 * (50.0–400.0)	0.7594
High (third)	163.5 ± 126.9	100.0 * (50.0–600.0)	175.9 ± 116.2	150.0 * (0.0–500.0)	0.4121
*p*-Value	0.8890		0.2736		

* distribution different than normal (verified using Shapiro-Wilk test—*p* ≤ 0.05).

**Table 2:** Vegetable intake (g/day) for boys and girls in Food Neophobia Scale categories—mean ± SD, as well as median, minimum, and maximum values are presented, and compared between genders and between food neophobia categories.

Food Neophobia Category (Tertile of Food Neophobia Scale)	Girls; *n* = 78		Boys; *n* = 85		*p*-Value
	Mean ± SD	Median (Minimum–Maximum)	Mean ± SD	Median (Minimum–Maximum)	
Low (first)	165.4 ± 119.0	100.0 * (0.0–400.0)	158.2 ± 107.3	100.0 * (50.0–500.0)	0.9635
Medium (second)	167.3 ± 103.9	200.0 * (0.0–400.0)	161.2 ± 144.8	100.0 * (50.0–800.0)	0.3796
High (third)	118.8 ± 105.2	100.0 * (0.0–500.0)	97.5 ± 76.4	100.0 * (0.0–300.0)	0.4988
*p*-Value	0.0144		0.0983		

* distribution different than normal (verified using Shapiro-Wilk test—*p* ≤ 0.05).

**Table 3:** Fruit and vegetable juice intake (g/day) for boys and girls in Food Neophobia Scale categories—mean ± SD, as well as median, minimum, and maximum values are presented, and compared between genders and between food neophobia categories.

Food Neophobia Category (Tertile of Food Neophobia Scale)	Girls; *n* = 78		Boys; *n* = 85		*p*-Value
	Mean ± SD	Median (Minimum–Maximum)	Mean ± SD	Median (Minimum–Maximum)	
Low (first)	115.4 ± 106.6	89.3 * (0.0–428.6)	195.8 ± 225.0	107.1 * (0.0–1000.0)	0.3245
Medium (second)	140.1 ± 119.0	107.1 * (0.0–357.1)	153.9 ± 147.9	107.1 * (0.0–535.7)	0.7998
High (third)	136.7 ± 185.2	71.4 * (0.0–642.9)	154.3 ± 127.5	142.9 * (0.0–500.0)	0.1496
*p*-Value	0.9602		0.5550		

* distribution different than normal (verified using Shapiro-Wilk test—*p* ≤ 0.05).

**Table 4:** Analysis of correlation between food neophobia level and fruit intake, vegetable intake, as well as fruit and vegetable juice intake.

	Girls; *n* = 78		Boys; *n* = 85	
	*p*	R	*p*	R
Fruits	0.069	−0.2071 *	0.842	−0.0219 *
Vegetables	0.032	−0.2432 *	0.004	−0.3071*
Fruit and vegetable juices	0.416	−0.0933 *	0.490	−0.0759 *

* Spearman’s rank coefficient.
